# Morphological and Pigment Responses to Far-Red and Photosynthetically Active Radiation in an Olive Cultivar Suitable for Super-High-Density Orchards

**DOI:** 10.3390/plants13131822

**Published:** 2024-07-02

**Authors:** Federico J. Ladux, Carina V. González, Eduardo R. Trentacoste, Peter S. Searles, M. Cecilia Rousseaux

**Affiliations:** 1 Centro Regional de Investigaciones Científicas y Transferencia Tecnológica de La Rioja (CRILAR-Provincia de La Rioja-UNLaR-SEGEMAR-UNCa-CONICET), Entre Ríos y Mendoza s/n, Anillaco 5301, La Rioja, Argentina; federicoladux@conicet.gov.ar (F.J.L.); psearles@conicet.gov.ar (P.S.S.); 2 Departamento de Ciencias Exactas, Físicas y Naturales (DACEF y N), Universidad Nacional de La Rioja, Av. Luis M. De la Fuente s/n, Ciudad Universitaria de la Ciencia y de la Técnica, La Rioja 5300, La Rioja, Argentina; 3 Instituto de Biología Agrícola de Mendoza (IBAM), FCA UNCuyo—CONICET, Almirante Brown 500, Chacras de Coria, Luján de Cuyo 5505, Mendoza, Argentina; cgonzalez@mendoza-conicet.gob.ar; 4 Facultad de Ciencias Agrarias, Universidad Nacional de Cuyo, Almirante Brown 500, Chacras de Coria, Luján de Cuyo 5505, Mendoza, Argentina; 5 Instituto Nacional de Tecnología Agropecuaria (INTA), Estación Experimental Agropecuaria La Consulta, Mendoza 5567, Argentina; trentacoste.eduardo@inta.gob.ar

**Keywords:** Arbequina, leaf area, *Olea europaea*, red/far-red ratio, shade, stem elongation

## Abstract

Plant density is increasing in modern olive orchards to improve yields and facilitate mechanical harvesting. However, greater density can reduce light quantity and modify its quality. The objective was to evaluate plant morphology, biomass, and photosynthetic pigments under different red/far-red ratios and photosynthetically active radiation (PAR) combinations in an olive cultivar common to super-high-density orchards. In a greenhouse, young olive trees (cv. Arbequina) were exposed to low (L) or high (H) PAR with or without lateral FR supplementation (L+FR, L-FR, H+FR, H-FR) using neutral-density shade cloth and FR light-emitting diode (LED) modules. Total plant and individual organ biomass were much lower in plants under low PAR than under high PAR, with no response to +FR supplementation. In contrast, several plant morphological traits, such as main stem elongation, individual leaf area, and leaf angle, did respond to both low PAR and +FR. Total chlorophyll content decreased with +FR when PAR was low, but not when PAR was high (i.e., a significant FR*PAR interaction). When evaluating numerous plant traits together, a greater response to +FR under low PAR than under high PAR appeared to occur. These findings suggest that consideration of light quality in addition to quantity facilitates a fuller understanding of olive tree responses to a light environment. The +FR responses found here could lead to changes in hedgerow architecture and light distribution within the hedgerow.

## 1. Introduction

Olive (*Olea europaea* L.) is a traditional woody crop species in the Mediterranean Basin that has expanded its production both inside and outside the Mediterranean in the last several decades [1,2]. Currently, it is cultivated on about 12.7 million hectares worldwide [3], with mostly low tree density (i.e., approx. 100 trees ha^−1^). In order to increase yields and facilitate harvesting, orchard design has become increasingly modernized, including very dense orchards known as super high-density (SHD) hedgerow orchards (1000 to 2500 trees ha^−1^) [4,5]. As in other fruit trees and vines, greater olive tree density not only leads to a more rapid increase in photosynthetic leaf area and crop yield, but it eventually also increases shading within and between plant rows, which may shorten orchard longevity [6,7].

As individual trees grow, many aspects of the light environment are modified within orchard systems. A number of plant species can sense the proximity of near neighbors early in stand development prior to direct shading via photoreceptors called phytochromes [8,9,10,11]. Phytochromes can detect early horizontal changes in the red (R, 660 nm) to far-red (FR, 730) ratio (R/FR) based on R being preferentially absorbed by green leaf tissue and FR being reflected horizontally between neighbors [12,13]. At later stages of crop development, direct shading significantly reduces photosynthetically active radiation (PAR; 400–700 nm) in the lower portions of the crop canopy and further reduces the R/FR ratio [11,14]. In SHD olive orchards, inter-row alleys are maintained at approximately fixed distances through lateral pruning, but daily PAR values still decrease significantly at middle and lower heights along the hedgerow walls and inside the hedgerow [6]. A recent study also observed that the horizontal R/FR ratio was lower along the hedgerow wall in an SHD orchard (1666 trees ha^−1^) compared to the outer tree canopy in a high-density orchard (408 trees ha^−1^) [15].

Plants can cope with shade using different strategies that are most often categorized as shade avoidance or tolerance [11]. Although very little is known about light quality responses in woody species such as fruit tree orchards compared to annual crop stands [16], peach and cherry trees appear to detect low R/FR ratio early and increase shoot elongation rates to avoid shade [17]. In contrast, no morphological responses to early, lateral low R/FR signals were observed in grapevines, which would indicate some degree of shade tolerance [18]. Shade avoidance strategies are often considered undesirable in agricultural crops because an increase in carbon allocation to shoots may reduce the carbon available for photosynthetic leaf area and crop yield [11]. Moreover, shade tolerance via leaf morphological and physiological adjustments to low PAR, including a more horizontal leaf angle and greater individual leaf area in the lower canopy of dense planting systems, may be beneficial for PAR absorption [19,20].

Domesticated olive (*Olea europaea* L.) is a sclerophyllous species with small evergreen leaves having xerophytic features such as trichomes and small stomata that help to reduce water loss and maintain plant growth in arid and semi-arid regions [21]. Its wild relatives are common constituents of maquis and garrigue scrub formations of varying plant densities [22]. Today, hundreds of olive cultivars have been identified and are grown for oil and table olive consumption [23]. Yet, only a handful of cultivars such as cv. Arbequina are considered suitable for SHD hedgerow orchards due to their relatively compact plant architecture and consistent yields under high plant densities [24,25].

Available information from shading experiments in olive trees using neutral-density shade cloth to reduce PAR intensity indicates that leaf morphology and photosynthetic pigments respond to low PAR through more horizontal leaf angles, decreased specific leaf mass (SLM) and stomatal density, as well as increased individual leaf area and chlorophyll concentration [26,27,28]. Much less information is available concerning the R/FR responses of olive trees. A recent experimental study in young trees using special, laterally positioned mirrors to supplement FR under high, outdoor PAR conditions (i.e., similar to the early orchard light environment) found that FR supplementation decreased both individual leaf area and total plant leaf area in olive cv. Arbequina [29]. In contrast, cv. Arauco showed some increase in individual, but not total, leaf area due to FR supplementation. The main stem elongation was not affected by the lateral supplementation in either cultivar. To the best of our knowledge, there is no experimental evidence concerning how olive trees may respond to R/FR under low PAR conditions typical of later orchard development. 

The need to evaluate light quantity and quality combinations simultaneously has been proposed for crop species because responses to shading may not be easily understood from the study of either light quantity or quality alone [10]. In sunflower seedlings, hypocotyl elongation increased when exposed separately to either a low R/FR ratio or to low PAR, but this increase was much greater when reducing them simultaneously [30]. Also in soybean, leaf area increased more when low R/FR was combined with low PAR, as did total chlorophyll content [31]. However, plant height decreased in the same study when low R/FR was combined with low PAR, but it increased when combined with high PAR. The responses of perennial crop and ornamental species to the combination of light quantity and quality remain largely unexplored [32]. 

The objective of this study was to evaluate plant morphology, biomass, and photosynthetic pigments under different R/FR ratios and PAR combinations in the olive cv. Arbequina, which is common in modern, super high-density orchards. The four combinations assessed were low PAR (L) with or without lateral FR supplementation (L+FR, L-FR) and high PAR (H) with or without lateral FR supplementation (H+FR, H-FR). The intensity of PAR was manipulated using neutral-density shade cloth, while FR supplementation was obtained using light-emitting diode (LED) modules. The experiment was performed for three months under greenhouse conditions using own-rooted 18-month-old young olive trees with an average main stem length of 30 cm at the beginning of the experiment.

## 2. Results

**Figure 1:**
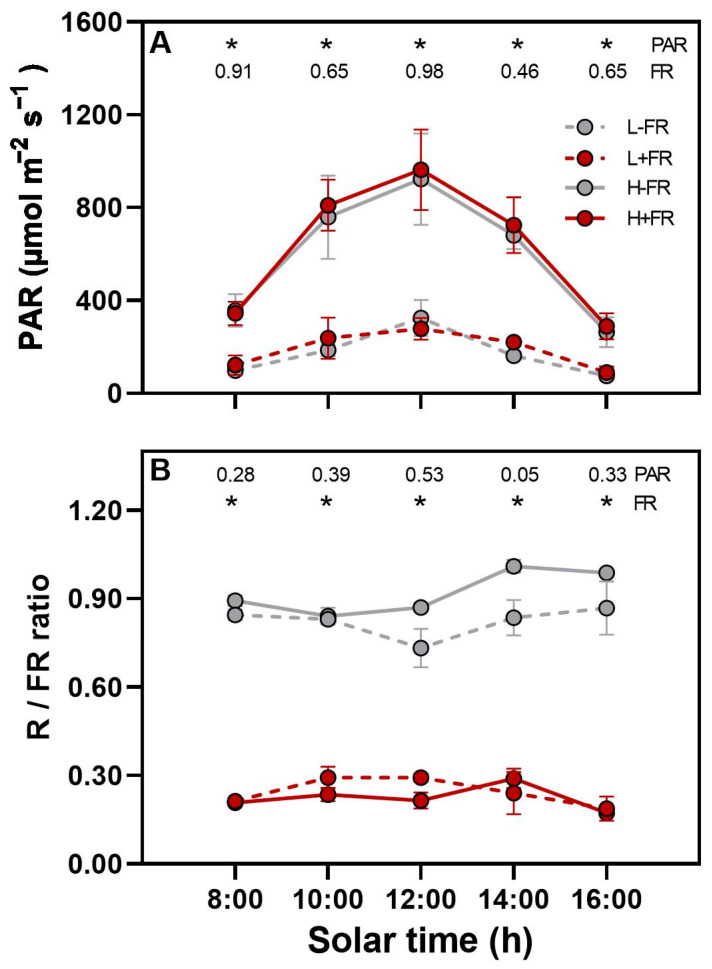
Diurnal light environment at the main stem apex for the different photosynthetically active radiation (low PAR, L; high PAR, H) (**A**) and lateral far-red (+FR, -FR) (**B**) combinations. PAR measurements were made with the sensor oriented upwards under neutral-density shade cloth or netting, while red/far-red (R/FR) ratios were measured horizontally towards the light-emitting diodes of the lateral +FR treatment or the dummy modules of the -FR control. The symbols represent averages ± SE (n = 4). Asterisks indicate significant differences between PAR levels in (**A**) and between +FR and -FR in (**B**) for a given solar time using Tukey’s post-test (*p* < 0.05). The numerical *p*-value is given when no significant difference occurs for PAR or FR. There were no significant interactions between PAR and FR.

As would be expected, the PAR at the main stem apex with the sensor oriented upwards was significantly less under L than under H throughout the day, with maximum average values at solar noon being approximately 300 and 1000 µmol m^−2^ s^−1^ in L and H, respectively, with no differences in PAR between the FR+ treatment and FR- control (Figure 1A). In a similar manner, the daily integrated PAR was estimated to be 6.8 mol m^−2^ day^−1^ in L and 23.0 mol m^−2^ day^−1^ in H. The lateral R/FR ratio at the main stem apex when the LED module faced south was much lower for the +FR treatment than for the -FR control under both PAR levels, with average daily values of 0.23 (+FR) and 0.87 (-FR) (Figure 1B). To further characterize the light environment, no significant differences in the lateral PAR were observed in any azimuth direction for a given PAR level. Lateral PAR near solar noon was 56 under L and 238 µmol m^−2^ s^−1^ under H. Lastly, the lateral R/FR ratio in the north, east, and west directions was about 0.94 for both +FR and -FR plants.

There were no differences in the average temperatures between PAR levels during the day (27.2 °C) or at night (17.9 °C) for the 6-day measurement period. The daily maximum and minimum temperatures were about 32 and 14 °C, respectively, during this period. Such values are considered to be representative of the overall experiment, given a comparison with outdoor temperatures from a nearby weather station.

### 2.1. Plant Morphology and Biomass

**Figure 2:**
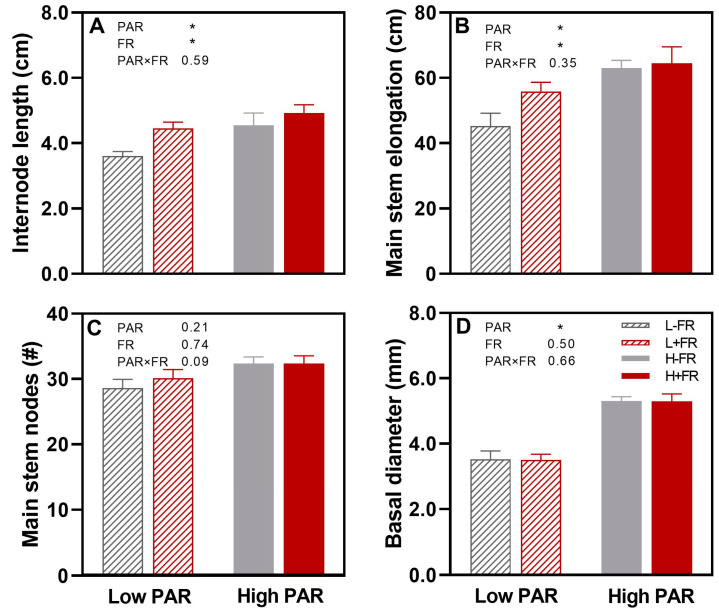
Internode length (**A**), stem elongation (**B**), stem nodes number (**C**), and basal diameter (**D**) for the main stem of olive cv. Arbequina plants exposed to different photosynthetically active radiation (low PAR, L; high PAR, H) and lateral far-red (+FR, -FR) combinations. Averages ± SE (n = 8) are shown for each treatment combination. The statistical probability level for PAR, lateral FR, and their interaction (PAR×FR) are given as the numerical *p*-value when not significant (*p* > 0.05, or * *p* < 0.05).

The main stem internode length and stem elongation were significantly less under L than under H (Figure 2A,B). In contrast, internode length and stem elongation were greater in the +FR plants than in the -FR control plants, with about a 20% increase under L and a lesser increase (<5%) under H. The number of main stem nodes was not affected by either PAR or FR supplementation (Figure 2C), while the basal diameter of the first new internode formed after the start of the experiment was 35% smaller under L than under H, with no FR supplementation effect (Figure 2C,D).

Similar to the main stem internode length, the axillary internode length was significantly less under L than under H, but it was greater due to FR supplementation (Table 1). Axillary internode length was 20% greater with +FR than with -FR under L and 7% under H. There were fewer axillary shoots and less total axillary shoot length under L than under H, but no statistically significant differences were apparent due to +FR. The axillary shoot insertion angle was not significantly affected by PAR. However, axillary shoots were more vertical (i.e., a lower shoot angle with respect to the main stem) under +FR than under -FR with an 11° difference between +FR and -FR under L and a 4° difference under H. PAR did affect leaf insertion angle on the main stem, with the angle being greater under L (86°) than H (78°), which indicates more horizontal leaves under L. Similar to the axillary shoot insertion angle, FR supplementation led to more vertical leaves at both PAR levels.

**Figure 3:**
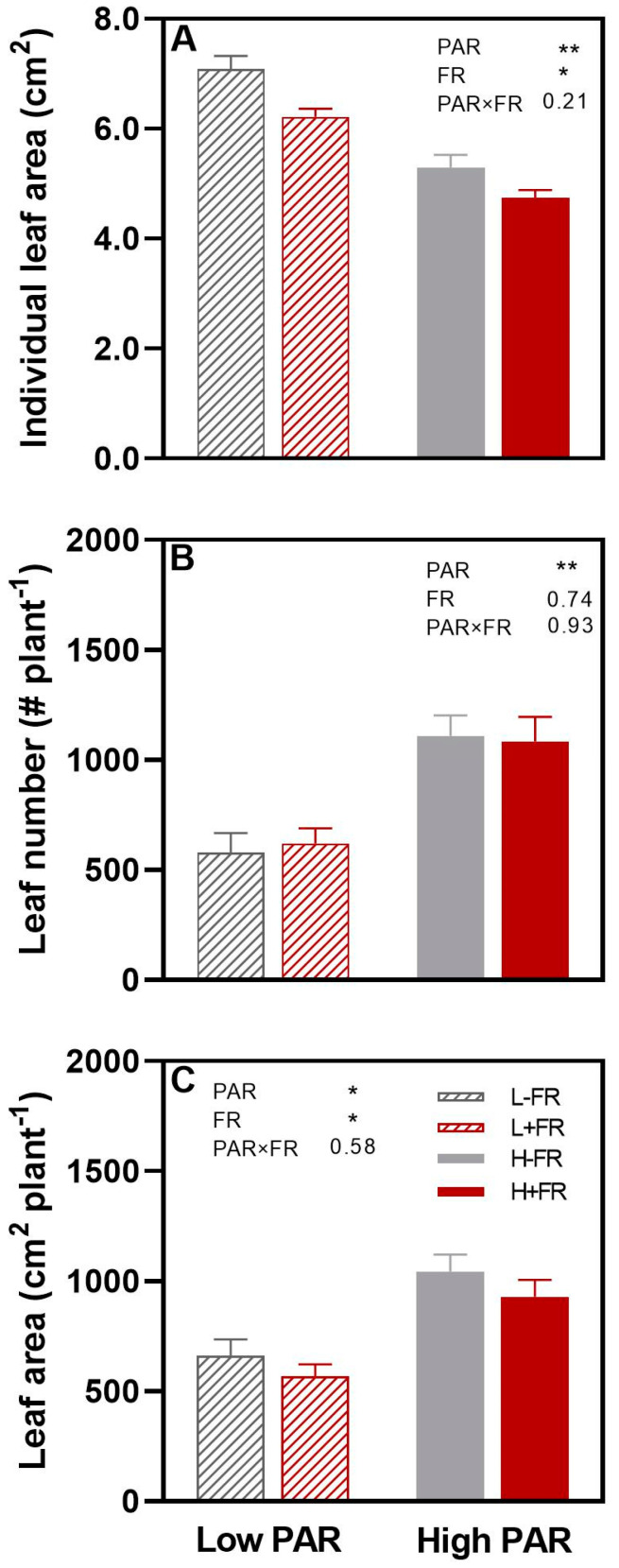
Individual leaf area (**A**), increase in leaf number per plant (**B**), and increase in leaf area per plant (**C**) of olive cv. Arbequina plants exposed to different photosynthetically active radiation (low PAR, L; high PAR, H) and lateral far-red (+FR, -FR) combinations. Averages ± SE (n = 8) are shown for each treatment combination. The statistical probability level for PAR, lateral FR, and their interaction (PAR×FR) are given as the numerical *p*-value when not significant (*p* > 0.05, * *p* < 0.05, or ** *p* < 0.01).

Individual leaf area was significantly greater (32%) under L than under H, while FR supplementation reduced individual leaf area by about 15% (Figure 3A). The increase in leaf number per plant during the experiment was much less under L than under H but was not affected by +FR (Figure 3B). On a plant basis, the leaf area increase during the experiment was about 40% less under L than under H due to fewer leaves being produced under L, while the leaf area increase was 14% less with +FR compared to -FR due to the lower individual leaf area (Figure 3C).

The leaf, stem, root, and total plant biomass were all more than 50% less under L than under H, with no response to FR supplementation (Table 2). However, the ratio of above-/below-ground biomass was greater under L than under H. Additionally, a greater specific stem length (cm g^−1^) and lower stem mass ratio [stem/(leaf + stem)] under L than under H indicate further changes in biomass allocation. In contrast, the stem mass ratio increased somewhat with +FR. Lastly, the leaf area ratio (total leaf area/leaf mass) was significantly greater under L than under H, while it was less under +FR than under -FR control.

### 2.2. Leaf Stomata and Photosynthetic Pigments

The stomatal conductance of water vapor was significantly less (−16%) under L than under H (Table 3). Low PAR leaves also had a lower stomatal density and length than H leaves. In contrast, no differences were apparent due to +FR. Chlorophyll a, chlorophyll b, total chlorophyll, and carotenoids were all greater under L than under H when expressed on a mass basis (Table 4). Interestingly, chlorophyll a and the total chlorophyll content were lower with +FR supplementation than in the -FR control under low PAR (L+FR versus L-FR), but no difference due to +FR was observed at high PAR (H+FR versus H-FR). This indicates a significant PAR×FR interaction term. In contrast, no statistically significant response related to +FR was observed in chlorophyll b or carotenoids. Additionally, no differences in the Chla:Chlb ratio were found between PAR or FR levels. 

### 2.3. Cluster and Principal Component Analyses

**Figure 4:**
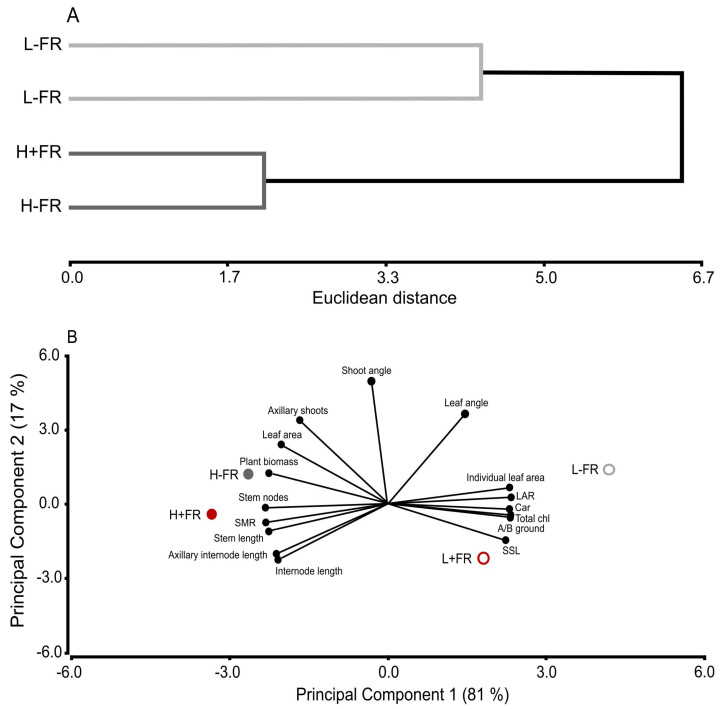
Cluster (**A**) and principal component (**B**) analyses of plant morphology, biomass, and photosynthetic pigment responses to different photosynthetically active radiation (low PAR, L; high PAR, H) and lateral far-red (+FR, -FR) combinations. The variables used in the analyses are individual leaf area, leaf area ratio (LAR), carotenoids (Car), total chlorophyll (Total chl), above-/below-ground (A/B ground) biomass ratio, specific stem length (SSL), internode length, stem elongation (stem length), stem nodes, plant biomass, stem mass ratio (SMR), leaf area per plant (leaf area), axillary internode length, number of axillary shoots (axillary shoots), axillary shoot angle (shoot angle), and leaf angle.

The cluster analysis shows that the responses of morphology, biomass, and photosynthetic pigment variables are grouped separately by L and H (Figure 4A). Furthermore, the greater Euclidean distance of the L grouping (4.3) than the H grouping (2.1) indicates more of an overall difference between +FR supplementation and the -FR control within the L grouping than in the H grouping. This greater overall difference between +FR and -FR under L occurred despite individual responses to +FR most often not being statistically different between PAR levels (i.e., a significant PAR×FR interaction term). In the principal component analysis (PCA), the first principal component (PC1) explained 81% of the variability, and the second principal component (PC2) explained 17%. (Figure 4B). On PC1, H was associated with a high number of axillary shoots (#), leaf area per plant, total plant biomass, main stem nodes, stem mas ratio (stem/(leaf + stem)), and stem elongation and internode length. In contrast, on PC1, L was associated with greater individual leaf area, leaf area ratio (total leaf area/leaf mass), carotenoids, and total chlorophyll on a mass basis, and above-/below-ground biomass along with specific stem length (main stem length/main stem mass). Lastly, the overall characteristics of +FR plants compared to the -FR controls were more different under L than under H, based on their average positions in Figure 4B. 

## 3. Discussion

The increase in plant density in olive tree orchards to enhance productivity and facilitate mechanical harvesting has gained renewed interest in the evaluation of tree responses to light quantity and quality [4,33]. The limited available information in olive orchards indicates that increasing plant density can decrease the lateral R/FR ratio between rows to about 0.4 [15] and that the vertical, downward R/FR ratio reaches minimum values of 0.2 within the lower canopy layers [34]. Detailed field measurements and simulation modeling of PAR in super high-density hedgerow orchards has found that PAR can be less than one-tenth of full sunlight in the lower canopy layers [7,15,35]. The current study under greenhouse conditions emulated such orchard light environments with the R/FR ratio of about 0.25 using lateral FR supplementation (LEDs) and downward PAR being reduced to less than 20% of outdoor values using neutral PAR shade cloth. As expected, it was found that low PAR reduced the total plant biomass and that of individual organs, while FR had no effect on biomass. However, several plant morphological variables, including main stem elongation, internode length, axillary internode length, individual leaf area, and leaf angle, responded to both low PAR and low R/FR. In some cases, the responses were opposite, such as shorter internodes under low PAR and longer internodes under +FR. Furthermore, a large collection of variables showed a greater response to +FR under low PAR than under high PAR when evaluated together based on the cluster and principal component analyses. For these reasons, consideration of light quality in addition to PAR facilitates a fuller understanding of plant responses in the lower, more shaded parts of olive tree orchards, as will be described further below.

Stem elongation is important because plant height strongly modifies the light environment during early hedgerow formation, and elongation at later stages also affects the alley width between rows. In the current study, main stem elongation was less under low than under high PAR, likely due to less photoassimilate availability associated with lower leaf net photosynthesis rates and photosynthetic capacity under low PAR [27,36,37]. Both midday and daily integrated PAR were about 70% less under L than under H, with midday values not exceeding 300 µmol m^−2^ s^−1^ under L (i.e., about 15% of full sunlight). As also occurred with internode length, FR supplementation increased main stem elongation, with the increase on a percentage basis being about 20% under L+FR compared to L-FR and a lesser increase (<5%) under H+FR compared to H-FR. The tendency for a greater effect of +FR under low PAR may be explained by the modulation of several photoreceptors, including phytochrome, cryptochrome, and UV8 [9]. Thus, with L+FR, both phytochrome and cryptochrome would not block the action of the phytochrome interacting factors (PIFs) that promote stem elongation. Also, it has recently been proposed that the B-box transcription factor BBX28 could promote growth under prolonged shade through the phytochrome system by perceiving the reduction of red photons [38]. Consistent with the tendency for a greater effect of +FR under low PAR found in our study, longer stems were observed only in a super-high-density compared to a high-density orchard of cv. Genovesa when R/FR and PAR were both low along the lower portion of the hedgerow wall in the super-high-density orchard [15]. Furthermore, little or no response of main stem elongation to FR supplemented using lateral mirrors under high PAR outdoor conditions was found in three olive cultivars, including cvs. Arbequina, Coratina, and Arauco [29]. Thus, in the few olive cultivars evaluated up until now, potential shading does not seem to be anticipated through phytochrome detection of low R/FR signals when PAR is still high. In contrast, increases in the main stem elongation of young peach and cherry trees were observed even due to modest decreases in the R/FR ratio under high PAR [17].

Tree branch architecture has been suggested to be critical when evaluating olive cultivar suitability for super-high-density orchards [39]. An evaluation of more than 20 mostly Italian cultivars found that cvs. Arbequina and Arbosana produce a greater number of lateral shoots than other cultivars and that these shoots have a smaller basal diameter [25]. These characteristics are advantageous for mechanical harvesting because more branching tends to increase fruit number within the physical dimensions of the harvester, and smaller diameters result in more flexible shoots. In the present study, low PAR led to fewer axillary shoots and less total axillary shoot length than high PAR, with no apparent response of these variables to +FR. Also, specific stem length was greater under L than under H, indicating somewhat longer and thinner shoots. In contrast, +FR plants had more vertically oriented axillary shoots than the -FR controls by 11° under low PAR and 4° under high PAR, while the axillary shoot angle did not differ significantly by PAR level. Although the shoot insertion angle has been reported to be similar between cv. Arbequina and other cultivars [25], the cv. Arbequina response to +FR supplementation has previously been unknown.

Leaf morphological attributes and pigments in olive trees have often been shown to be affected by low PAR, including increased individual leaf area, more horizontal leaf angles, and greater chlorophyll concentrations [26,27,28]. Such responses have been shown to improve PAR interception in tree canopies and evergreen plant species communities [40,41]. However, little or no information is available concerning +FR responses. In the present study, individual leaf area was increased under low PAR, but was reduced by +FR supplementation possibly due to a greater carbon allocation to internode growth than to leaf growth, as observed in several other species [9]. Leaf angle also showed different responses to low PAR and +FR, with more horizontal leaves being found under low PAR and more vertical leaves under +FR supplementation. Leaf angles are highly responsive to PAR intensity, with more horizontal leaves under low PAR improving light capture. On the other hand, more vertical leaves under +FR have been shown to optimize leaf position within canopies and to occur in anticipation of shading by neighbors [9]. It has previously been suggested for *Arabodopsis thaliana* that phytochrome interacting factors (PIFs) are involved in these responses [42]. In our previous outdoor study under high PAR, FR supplementation was also associated with smaller leaves in cv. Arbequina [29]. FR supplementation has been found to lead to more vertical leaf inclination angles in *Rosa hybrida* stands, although the individual leaf area was not affected by FR [32]. Thus, leaf responses are likely to differ by species, and possibly within species as well. Interestingly, total chlorophyll on a mass basis decreased in the present study under +FR when PAR was low, but not when PAR was high (i.e., a significant FR*PAR interaction). In a somewhat similar manner, leaf senescence was induced in sunflower when FR supplementation was combined with low but not with high PAR [43], which suggests that chlorophyll synthesis and degradation is regulated by several photoreceptors, including phytochromes and cryptochromes, that integrate a response to a given light environment [44]. Overall, more vertical axillary shoot and leaf insertion angles, along with smaller leaves of lower chlorophyll content due to +FR, could affect PAR distribution in olive hedgerows. It may be suggested that downward PAR would penetrate further into the lower hedgerow canopy, where PAR is limiting. However, vertically oriented shoots and leaves may not improve horizontal PAR penetration into the hedgerow wall.

As would be expected, total plant biomass and that of individual organs were much less under low PAR than under high PAR in the current study. Of greater interest, FR supplementation did not affect the total biomass in cv. Arbequina, as was also found in [29] for the same cultivar under high PAR. However, [29] reported an apparent reduction (−20%) in plant biomass in cv. Coratina due to +FR under high PAR, which suggests that cultivar differences should be further explored. In the current study, total biomass was not affected by +FR supplementation under low PAR, although the total plant leaf area and leaf chlorophyll content were lower under this treatment. This suggests that leaf gas exchange measurements would prove helpful in future studies to assess leaf net photosynthesis rates under +FR. Low R/FR ratios have been shown to increase the leaf net photosynthetic rate under some conditions in soybean [31]. Under shaded conditions, plants generally allocate more biomass to stem elongation at the expense of leaf and root development [11,45,46]. In the current study, biomass partitioning was affected by both PAR and FR, including a greater above-/below-ground ratio under low PAR than under high PAR and more allocation to stems than leaves due to +FR. In contrast, the above-/below-ground ratio was not influenced by low PAR in the young plants of another common olive hedgerow cultivar (cv. Arbosana) [26]. More allocation to stems than to leaves with +FR concurs with the greater main stem elongation and smaller individual leaf area found with +FR. Some previous studies with other species have proposed that inhibition of leaf expansion under +FR may occur due to competition for photoassimilates between leaf and stem growth [47,48].

To better understand lateral FR responses in hedgerows, it must be asked whether the responses are the same across a wide range of PAR or if they differ by PAR quantity. Thus, the design of this study included +FR and -FR in combination with both low and high PAR levels. Despite individual responses to +FR most often not being statistically different between PAR levels (i.e., a significant PAR×FR interaction term), a greater response to +FR under low PAR than under high PAR was observed when evaluating a large number of morphology, biomass, and pigment variables together based on the cluster and principal component analyses. These results indicate that +FR led to a lower individual leaf area, leaf area ratio, and total chlorophyll content under low PAR than under high PAR. Soybean plant height, leaf area, and chlorophyll content were also found to respond differently to the R/FR ratio under low and high PAR, although the specific responses were variable-dependent [31]. Different sensitivities of plant variables to the R/FR ratio under low than under high PAR are likely explained by one or several photoreceptors acting together (UV-B photoreceptors, phototropins, cryptochromes, phytochromes) when strong signals are provided under heavily shaded conditions [49,50,51].

## 4. Materials and Methods

### 4.1. Plant Material and Growing Conditions

The experiment was performed in a greenhouse from mid-spring to early summer (1 November 2019 to 24 January 2020) at the INTA-Junín experimental field station in Mendoza, Argentina (33°6′ S; 68°29′ W; 653 m above sea level). The greenhouse had a transparent polycarbonate roof and was cooled with an evaporative fan–pad system that included two large exhaust fans on one side of the greenhouse for expelling warm air and an evaporative pad on the other side to cool air entering the greenhouse. The plants (cv. Arbequina) were own-rooted and cultivated in the field station nursery from cuttings. The nursery was covered by shade cloth with a 50% neutral-density light transmission, and the plants were grown in 3 L plastic pots containing a sand–peat–perlite mixture (1:1:1). The plants were 18 months old at the beginning of the experiment, with an average main stem length of 30 cm and 23 main stem leaves. They were watered twice a day using a microtube spider-type irrigation system during the experiment and received a total of 9 g per plant of slow release, granulated NPK (16-8-12) containing micronutrients (B, Cu, Fe, Mn, Zn, Mo) (Basacote Plus 6M, Compo Expert, Santiago, Chile). Fertilization was done in three applications of 3 g each at 15-day intervals during the first half of the experiment.

### 4.2. Light Treatments and Experimental Layout

The four light treatments included low (L) or high (H) PAR with either supplemental lateral FR (+FR) or no FR supplementation (-FR). Thus, the treatments were low PAR with supplemental lateral FR (L+FR), low PAR with no supplemental lateral FR (L-FR), high PAR with supplemental lateral FR (H+FR), and high PAR with no supplemental lateral FR (H-FR). The PAR levels were obtained by covering rectangular frames above greenhouse benches with either a 25% PAR transmittance shade cloth (L) or a neutral wavelength density netting that transmitted 90% of the incident PAR (H). Half of each bench was covered by the 25% PAR shade cloth, and the other half by the 90% PAR netting. The maximum PAR intensity measured at midday on a sunny day was 300 and 1000 μmol m^−2^ s^−1^ for L and H, respectively. 

FR supplementation was applied to individual plants on the benches using light-emitting diodes (LEDs) with a peak emission at 730 nm (Green Power LED, Philips, Amsterdam, Netherlands). Each +FR plant had its own vertical LED module positioned 0.1 m from the plant on the southern side. A module contained five LEDs spaced at 0.1 m intervals in height, with the central LED located at the main stem apex. The supplementation was approximately 70 μmol m^−2^ s^−1^ during the entire natural photoperiod plus 1 h at the end of the day [18]. The photoperiod length was between 13:26 h and 14:19 h during the experiment. The +FR treatment duration was controlled using digital timers, which were programmed weekly to account for changing photoperiod length. For the -FR treatments, a black foam strip was used as a dummy to simulate the physical presence of an LED module.

At the beginning of the experiment, half of each of the two available greenhouse benches was designated as low PAR or high PAR, and each individual plant on a bench received either +FR or -FR as mentioned above. The benches were 6 m long and oriented east–west in the greenhouse. Eight plants were randomly assigned to each of the four treatments (i.e., n = 8 per treatment) and arranged in a staggered, zigzag formation with a spacing of 0.9 m between them along a bench. A rotation scheme was devised such that each plant was moved one position along a bench within its given PAR level every 10 days to reduce possible position effects within the greenhouse. By the end of the experiment, each plant had been positioned at all of the available positions on both benches for a given PAR level. Thus, an individual plant was considered to be an appropriate experimental replicate in a PAR×FR (2 × 2) factorial design.

### 4.3. Light and Air Temperature Measurements

The PAR (400–700 nm) and R/FR ratio (660/730 nm) were characterized within the greenhouse on one cloudless day (November 15). The measurements were made for two randomly selected plants per treatment on each bench. The PAR was determined at the main stem apex using a quantum sensor (model MQ-500r, Apogee Instruments, Logan, UT, USA) oriented upwards and also laterally towards the north, south, east, and west directions. The R/FR ratio was measured at the main stem apex using a hand-held sensor (Model SKR 110, Skye Instruments Ltd., Llandrindod Wells, UK) oriented laterally towards the LED module or black foam strip dummy to the south of each plant. Some additional lateral R/FR ratio measurements were performed towards the north, east, and west to further assess the R/FR environment. All measurements were performed five times over the course of the day (8, 10, 12, 14, and 17 h solar time). 

Air temperature was recorded on the first bench every 60 min for each PAR level using the built-in temperature sensor of one data logger (MX100, HOBO-Onset Computer Corporation, Bourne, MA, USA) over three days in early November. The data loggers were then moved to the second bench for three days of similar measurements. Average daytime, nighttime, and midday air temperatures were then calculated for each PAR level. These temperatures were compared to outdoor temperatures from an automatic weather station at the experimental field station to determine their representativeness. 

### 4.4. Plant Morphology and Biomass

The main stem length and the number of nodes and leaves on the main stem were measured at the beginning and end of the experiment to calculate the changes in these variables over the course of the 85-day experiment. Internode length and basal diameter of the first new internodes formed on the main stem during the experiment were also measured. Although there were few axillary shoots at the beginning of the experiment, their number and length were determined at the beginning and end of the experiment to calculate the increase in axillary shoot number and in the total axillary shoot length. Axillary internode length was estimated as the increase in total axillary shoot length per plant divided by the total number of axillary nodes formed during the experiment.

Leaf length and width were determined by randomly measuring 10 fully developed leaves per plant formed during the experiment on either the main stem or axillary shoot nodes. Individual leaf area was then obtained for these same 10 leaves after sampling one leaf disk of known area per leaf and drying the leaves and disks in a forced-air oven at 70 °C until constant weight was reached. The weight of the disks with their known leaf area was then used to calculate the area for an entire individual leaf. The increase in total plant leaf area was determined as the individual leaf area multiplied by the increase in leaf number per plant over the course of the experiment.

For all axillary shoots formed during the experiment, the axillary shoot insertion angle was measured as the angle between the main stem above the insertion point and the axillary shoot. The leaf insertion angle was similarly determined as the angle between the main stem above the leaf and the leaf itself. This angle was determined for 10 main stem leaves per plant. When comparing treatments, a larger axillary shoot or leaf insertion angle indicates a more horizontally oriented leaf, while a smaller angle represents a more vertically oriented leaf.

Each plant was harvested at the end of the experiment to determine the leaf, stem, and root biomass. The leaf and stem biomass includes both the main stem and axillary shoot biomass. The material was dried at 70 °C in a forced-air oven until it reached a constant dry weight. The above-/below-ground biomass ratio, specific stem length (main stem length/main stem mass), stem mass ratio [stem biomass/(leaf +stem mass)], and leaf area ratio (total leaf area/leaf mass) were also calculated.

### 4.5. Stomatal Conductance and Density

The stomatal conductance of water vapor of two fully expanded leaves per plant was measured during the mid-morning on a clear day towards the end of the experiment using a leaf porometer (model SC-1, Decagon Devices, Pullman, WA, USA). Stomatal density, stomatal length, and width were determined on one similar leaf using stomatal imprints. The imprints were taken from leaves collected before sunrise, when the stomates were closed, and kept in a darkened laboratory during the procedure. The trichomes were first removed with adhesive tape from the abaxial leaf surface, and then a thin layer of transparent nail polish was applied to the entire abaxial surface. Once dried, the imprints were peeled off the abaxial surface using a fine-tipped forceps. Ten images with a known area were captured per imprint under a compound microscope (400×, Leica QWin Software 3.1.0). From each image, the stomata number as well as the length and width of 10 stomata were determined using ImageJ software (1.52) (U.S. National Institutes of Health, https://imagej.nih.gov/ij; accessed on 9 March 2020).

### 4.6. Photosynthetic Leaf Pigments

Two fully expanded leaves per plant that developed during the experiment were sampled and kept in a cooler during transport from the greenhouse to the laboratory for chlorophyll and carotenoid determination. In the laboratory, two leaf disks of 1 cm^2^ per plant were incubated in the dark in 10 mL of dimethyl sulfoxide at 70 °C for 45 min and then subjected to sonication in an ultrasound tray at 60 °C for 15 min. The 45 min incubation and 15 min sonication cycles were repeated three times. Absorbance was then measured at 665, 649, and 480 nm in a spectrophotometer (model SP 2000 UV, Spectrum Instruments, Shanghai, China). Total chlorophyll (Chl) and carotenoids, as well as Chla and Chlb, were calculated according to the equations of [52]. 

### 4.7. Statistical Analyses

The individual plant response variables were analyzed using a linear mixed-effects model to determine the main effects of PAR and FR along with their potential interaction. The fixed factors in the model were PAR and FR, with the plant being a random factor. The main stem leaf number at the beginning of the experiment was used as a covariate for assessing treatment differences for the plant morphology and biomass variables. The most appropriate variance structure for each analysis was determined by comparing the Akaike and Bayesian information criteria.

Further analyses were performed in order to gain insight into the overall plant responses. For this reason, a hierarchical clustering analysis by average linkage (Euclidean distance) was performed to group the four light treatments based on the mean values of the morphology, biomass, and pigment variables. A principal component analysis was also used to identify potential patterns due to the light treatments for these same plant variables. All statistical analyses were performed using InfoStat statistical software (2020) [53].

## 5. Conclusions

Little attention has been given to the potential role of light quality in fruit tree orchards. The greenhouse study presented here with olive cv. Arbequina, a common cultivar in super-high-density hedgerow orchards, indicates that lateral FR supplementation affects plant morphological and biomass allocation traits as well as chlorophyll content more under low than under high PAR. In other words, the responses were greater under shaded conditions typical of mature hedgerow orchards than those of impending shade early in hedgerow formation. These responses likely have consequences for hedgerow architecture and light distribution within hedgerows. Further research is needed to evaluate these findings in more olive cultivars and to assess possible flowering responses to the R/FR ratio and blue light.

## Figures and Tables

**Table 1:** Axillary shoot characteristics and leaf angle of olive cv. Arbequina plants exposed to different photosynthetically active radiation (low PAR, high PAR) and lateral far-red (+FR, -FR) combinations. Averages ± SE (n = 8) are shown for each treatment combination. The statistical probability level for PAR, lateral FR, and their interaction (PAR×FR) are given as the numerical *p*-value when not significant (*p* > 0.05, * *p* < 0.05, and ** *p* < 0.01).

Variable	Low PAR		High PAR				
	-FR	+FR	-FR	+FR	PAR	FR	PAR×FR
Axillary internode length (cm)	2.0 ± 0.2	2.5 ± 0.1	2.6 ± 0.1	2.8 ± 0.1	*	*	0.09
Axillary shoots (number)	9 ± 2	5 ± 1	12 ± 2	12 ± 3	*	0.31	0.47
Total axillary shoot length (cm)	53 ± 14	55 ± 9.1	213 ± 20	203 ± 24	**	0.83	0.75
Axillary shoot angle (°)	71 ± 1	60 ± 2	71 ± 3	67 ± 1	0.09	*	0.09
Leaf angle (°)	95 ± 3	76 ± 2	86.± 5	69 ± 6	*	**	0.81

**Table 3:** Stomatal conductance, density, and dimensions of olive cv. Arbequina plants exposed to different photosynthetically active radiation (low PAR, high PAR) and lateral far-red (+FR, -FR) combinations. Averages ± SE (n = 8) are shown for each treatment combination. The statistical probability level for PAR, lateral FR, and their interaction (PAR×FR) are given as the numerical *p*-value when not significant (*p* > 0.05, or * *p* < 0.05).

Variable	Low PAR		High PAR				
	-FR	+FR	-FR	+FR	PAR	FR	PAR×FR
Stomatal conductance (mmol m^−2^ s^−1^)	223 ± 9	238 ± 16	283 ± 14	267 ± 18	*	0.97	0.31
Stomatal density (number mm^−2^)	216 ± 5	217 ± 6	244 ± 7	240 ± 11	*	0.72	0.84
Stomata length (μm)	22.0 ± 0.2	21.9 ± 0.4	22.7 ± 0.4	22.9 ± 0.3	*	0.91	0.79
Stomata width (μm)	15.7 ± 0.2	15.4 ± 0.3	16.0 ± 0.2	15.9 ± 0.2	0.13	0.30	0.75

**Table 4:** Photosynthetic leaf pigments of olive cv. Arbequina plants exposed to different photosynthetically active radiation (low PAR, high PAR) and lateral far-red (+FR, -FR) combinations. Averages ± SE (n = 8) are shown for each treatment combination. The statistical probability level for PAR, lateral FR, and their interaction (PAR×FR) are given as the numerical *p*-value when not significant (*p* > 0.05, * *p* < 0.05, and ** *p* < 0.01). Different letters indicate significant mean differences between PAR and FR combinations for a given plant variable using Tukey’s post-test (*p* < 0.05).

Variable	Low PAR		High PAR				
	-FR	+FR	-FR	+FR	PAR	FR	PAR×FR
Chlorophyll a (μg mg^−1^)	0.70 ± 0.01 a	0.62 ± 0.01 b	0.42 ± 0.04 c	0.43 ± 0.03 c	**	0.17	*
Chlorophyll b (μg mg^−1^)	0.29 ± 0.01	0.26 ± 0.03	0.18 ± 0.01	0.18 ± 0.01	**	0.13	0.08
Total chlorophyll (μg mg^−1^)	0.99 ± 0.02 a	0.88 ± 0.01 b	0.60 ± 0.05 c	0.61 ± 0.04 c	**	0.15	*
Carotenoids (μg mg^−1^)	0.11 ± 0.003	0.10 ± 0.004	0.08 ± 0.01	0.09 ± 0.01	*	0.34	0.21

**Table 2:** Biomass and related variables of olive cv. Arbequina plants exposed to different photosynthetically active radiation (low PAR, high PAR) and lateral far-red (+FR, -FR) combinations. Averages ± SE (n = 8) are shown for each treatment combination. The statistical probability level for PAR, lateral FR, and their interaction (PAR×FR) are given as the numerical *p*-value when not significant (*p* > 0.05, * *p* < 0.05, and ** *p* < 0.01).

Variable	Low PAR		High PAR				
	-FR	+FR	-FR	+FR	PAR	FR	PAR×FR
Leaves (g)	6.3 ± 0.7	6.0 ± 0.6	13.5 ± 0.5	12.4 ± 1.3	**	0.39	0.60
Stems (g)	3.6 ± 0.6	3.9 ± 0.4	10.4 ± 0.7	10.2 ± 1.1	**	0.91	0.76
Roots (g)	3.9 ± 0.3	4.3 ± 0.5	13.7 ± 0.9	13.8 ± 1.7	**	0.79	0.92
Above-/below-ground ratio	2.6 ± 0.2	2.4 ± 0.2	1.8 ± 0.1	1.7 ± 0.1	**	0.44	0.79
Total plant (g)	13.8 ± 1.6	14.2 ± 1.4	37.6 ± 1.6	36.5 ± 3.9	**	0.88	0.74
Specific stem length (cm g^−1^)	29.0 ± 2.5	29.7 ± 2.1	15.2 ± 0.7	17.3 ± 0.9	**	0.99	0.63
Stem mass ratio (stem/(leaf + stem))	0.36 ± 0.01	0.40 ± 0.01	0.43 ± 0.01	0.45 ± 0.01	**	*	0.29
Leaf area ratio (cm^2^ g^−1^)	55.5 ± 2.6	45.4 ± 1.3	30.8 ± 2.1	27.4 ± 2.0	**	*	0.11

## Data Availability

Data available from the authors on request.
